# Glutathione Protects against Paraquat-Induced Oxidative Stress by Regulating Intestinal Barrier, Antioxidant Capacity, and CAR Signaling Pathway in Weaned Piglets

**DOI:** 10.3390/nu15010198

**Published:** 2022-12-30

**Authors:** Xuan Xiang, Houfu Wang, Wentao Zhou, Chenyu Wang, Peng Guan, Gang Xu, Qiang Zhao, Liuqin He, Yulong Yin, Tiejun Li

**Affiliations:** 1 Hunan Provincial Key Laboratory of Animal Nutritional Physiology and Metabolic Process, CAS Key Laboratory of Agro-Ecological Processes in Subtropical Region, National Engineering Laboratory for Pollution Control and Waste Utilization in Livestock and Poultry Production, Institute of Subtropical Agriculture, Chinese Academy of Sciences, Changsha 410125, China; 2 College of Advanced Agricultural Science, University of Chinese Academy of Sciences, Beijing 100049, China; 3 Hunan Provincial Key Laboratory of Animal Intestinal Function and Regulation, Laboratory of Animal Nutrition and Human Health, College of Life Sciences, Hunan Normal University, Changsha 410081, China; 4 Hunan Flag Bio-Tech Co., Ltd., Changsha 410100, China

**Keywords:** glutathione, constitutive androstane receptor, weaned piglets, oxidative stress, intestinal barrier

## Abstract

Endogenous glutathione (GSH) effectively regulates redox homeostasis in the body. This study aimed to investigate the regulatory mechanism of different dietary levels of GSH supplementation on the intestinal barrier and antioxidant function in a paraquat-induced stress-weaned piglet model. Our results showed that dietary 0.06% GSH supplementation improved the growth performance of weaned piglets under normal and stressful conditions to some degree and decreased the diarrhea rate throughout. Exogenous GSH improved paraquat-induced changes in intestinal morphology, organelle, and permeability and reduced intestinal epithelial cell apoptosis. Moreover, GSH treatment alleviated intestinal oxidative stress damage by upregulating antioxidant (*GPX4*, *CnZnSOD*, *GCLC*, and *GCLM*) and anti-inflammatory (*IL-10*) gene expression and downregulating inflammatory cytokines (*IFN-γ* and *IL-12*) gene expression. Furthermore, GSH significantly reduced the expression levels of *constitutive androstane receptor (CAR)*, *RXRα*, *HSP90*, *PP2Ac*, *CYP2B22*, and *CYP3A29*, and increased the expression levels of *GSTA1* and *GSTA2* in the jejunum and ileum of paraquat-induced piglets. We conclude that exogenous GSH protects against oxidative stress damage by regulating the intestinal barrier, antioxidant capacity, and CAR signaling pathway.

## 1. Introduction

In healthy animals, the concentrations of reactive oxide species (ROS) are in a state of dynamic equilibrium, whereas when the animals are stimulated by internal and external factors, an excess of free radicals is formed and accumulated in the body, causing oxidative stress and antioxidant dysfunction [1]. It has been reported that oxidative stress disrupts the intestinal morphological structure, tight junctions, permeability, and gut microbiota of weaned piglets, which, in turn, affects the digestion and absorption, material metabolism, and immune function of the organism [2]. Exogenous supplementation of some nutrients or antioxidants, such as functional amino acids, mineral elements, vitamins, plant extracts, and probiotics, can regulate the intestinal barrier function and redox balance of animals to relieve oxidative stress [3,4,5]. Additionally, the exogenous addition of nutrients and antioxidants can provide raw materials for glutathione (GSH) synthesis and increase its levels in the body, which improves antioxidant capacity and immune function [6].

The most important of these antioxidant systems in the organism is GSH, which plays a key role in maintaining a dynamic cellular redox balance [7]. Endogenous GSH can help the body scavenge free radicals and peroxides by combining its sulfhydryl groups with free radicals and promoting the synthesis of antioxidant enzymes, such as superoxide dismutase (SOD), thus maintaining relative stability in the animal body [8]. It is also a broad-spectrum detoxifying agent that can directly combine with some heavy metal ions, carcinogenic substances, aflatoxin, and other toxic substances, which are excreted from the body under the action of enzymes [9]. Animal studies have shown that GSH improves semen preservation in males, protects follicle development in females, and improves the antioxidant function of the animal body, promoting growth and enhancing the resistance of animals to adverse environmental effects [10,11,12,13].

The constitutive androstane receptor (CAR) is a lipid-soluble ligand-dependent transcription factor that regulates drug metabolism in vivo and plays a major role in gluconeogenesis, lipid metabolism, bile acid metabolism, and apoptosis [14,15]. Furthermore, CAR regulates the expression of antioxidant genes (e.g., SOD, catalase (CAT), and glutathione peroxidase (GSH-PX)) and detoxification-encoding genes (e.g., cytochrome P450 (CYP450), and glutathione-S-transferases (GSTs)) in response to ROS, thereby resisting oxidative stress caused by adverse internal and external stimuli [15,16,17]. Excessive intake of acetaminophen is converted to N-acetyl-p-benzoquinone imine by CYP450, which depletes GSH and causes oxidative stress and hepatotoxicity; the whole process is apparently dependent on CAR [18]. This suggests that there may be some correlation between GSH and CAR signaling; nevertheless, the regulatory role of GSH in the CAR signaling pathway has not been studied in detail. In addition, GSH has more applications in aquatic animals and ruminants than in weaned piglets. Therefore, this study was conducted to investigate whether exogenous supplementation of GSH in the diet can improve the growth performance and antioxidant capacity of weaned piglets and its underlying mechanism of action.

## 2. Materials and Methods

All animal experiments used in this study were approved by the Animal Welfare Committee of the Institute of Subtropical Agriculture, Chinese Academy of Sciences (protocol code 20220056 of 21 June 2022; Changsha, China).

### 2.1. Experimental Materials

Glutathione (purity ≥ 95.51%) was obtained from Hunan Flag Bio-tech (Changsha, China). Paraquat was purchased from Chengdu Huaxia Chemical Reagent (Chengdu, China). Three-breed crossbred (Duroc × Landrace × Yorkshire) weaned piglets were purchased from Hunan Longhua Animal Husbandry Development (Zhuzhou, China).

### 2.2. Animal and Experimental Design

**Figure 1:**
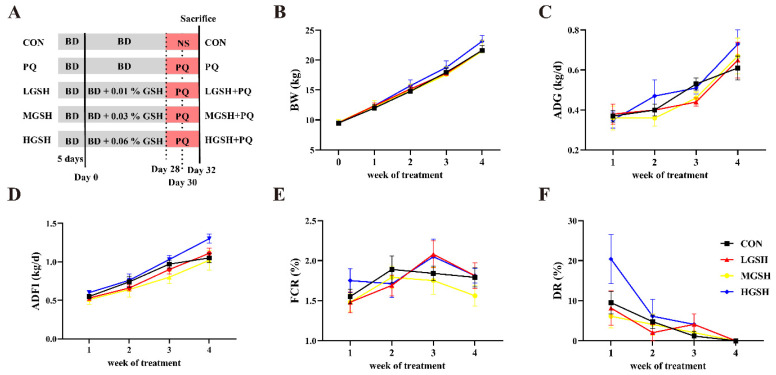
Effect of dietary GSH supplementation at different levels on growth performance in weaned piglets. (**A**) Experimental design and timeline (BD—basal diet; NS—normal saline). (**B**–**F**) The BW, ADG, ADFI, FCR, and DR per week for different groups. Data values with different small letter superscripts indicate a significant difference (*p* < 0.05), while with the same or no letter superscripts indicate no significant difference (*p* > 0.05). Values are expressed as mean ± SEM, *n* = 7.

Thirty-five healthy weaned piglets (Duroc × Landrace × Yorkshire) with similar body weight (BW = 9.52 ± 0.20 kg) were randomly assigned to 5 groups (*n* = 7/group) as follows: control (CON) and paraquat (PQ) groups were fed a basal diet, and GSH treatment groups were fed experimental diets with 0.01% GSH (LGSH), 0.03% GSH (MGSH), and 0.06% GSH (HGSH). The ingredient and nutrient levels of the basal diets without antibiotics or antioxidants met the nutrient specifications for pigs with a BW of 10–30 kg according to the recommendations of the NRC (2012) (Table 1). After 5 days of adaptation, GSH was added to the basal diet according to the corresponding dose every day, which was directly mixed into the feed once in the morning. The whole experiment lasted for 33 days; after 4 weeks of feeding, piglets in the PQ group and different GSH groups were intraperitoneally injected with paraquat at a dose of 8 mg/kg BW on days 28, 30, and 32. The CON group was injected with the same volume of saline until slaughter on day 33 (the experiment procedure is shown in Figure 1A). During the experiment, the piglets were housed individually and provided free access to water. Feed intake and diarrhea were recorded daily, BW was recorded weekly, and weekly average daily feed intake (ADFI), average daily gain (ADG), feed conversion ratio (FCR), and diarrhea rate (DR) were calculated from the records. The calculation formula is as follows: ADFI = total feed intake/days; ADG = (initial BW − final BW)/days; FCR = ADFI/ADG; DR = number of diarrhea/(number of pigs per group × days) × 100%.

### 2.3. Sample Collection

After fasting for 12 h, blood samples were collected from the anterior vena cava on the morning of the 33rd day of the experiment and were placed in common blood collection vessels. The blood was centrifuged at 3000 r/min for 10 min at 4 °C, and serum was collected. At the end of the experiment, all piglets were slaughtered by carotid exsanguination, and the abdominal cavity was quickly opened to isolate the jejunum and ileum. The jejunal and ileal tissues (approximately 1 cm) were immediately fixed in 4% paraformaldehyde and 3% glutaraldehyde for histological analysis. The parts of jejunum and ileum were collected, then rapidly frozen in liquid nitrogen and stored at −80 °C.

### 2.4. Serum Physiological and Biochemical Properties

Serum malondialdehyde (MDA), total antioxidant capacity (T-AOC), CAT, and SOD levels were determined using colorimetric assay kits (Beijing Boxbio Science & Technology, Beijing, China) as previously reported according to the manufacturer’s instructions [19]. Serum GSH-PX, GSH, oxidized glutathione (GSSG), diamine oxidase (DAO), and intestinal fatty acid binding protein (iFABP) levels were measured using ELISA kits from Jiangsu Meimian Industrial (Jiangsu, China).

### 2.5. Intestinal Histomorphology

The jejunal and ileal samples were collected, fixed in 4% paraformaldehyde, dehydrated, embedded in paraffin, and sliced into 6 µm sections. After hematoxylin and eosin staining, the sections were dehydrated and sealed. Six good visual fields were selected to measure villus height and crypt depth, and the villus height/crypt depth ratio was calculated as in a previous study [20]. Histological changes were observed using a fluorescence microscope (BX51; Olympus, Tokyo, Japan).

### 2.6. Cell Apoptosis

Apoptosis was detected using a one-step TUNEL apoptosis assay kit (Beyotime Biotech, Shanghai, China). Paraffin sections were dewaxed with xylene, anhydrous ethanol, 90% ethanol, 70% ethanol, and distilled water, then reacted with 20 µg/mL of DNase-free Proteinase K for 30 min at 25 °C, followed by 60 min at 37 °C with the configured TUNEL reaction solution and washed twice with phosphate-buffered saline. After blocking the slices, the fluorescence signals were observed by fluorescence microscope (DM3000; Leica, Shanghai, China), and apoptotic cells were counted.

### 2.7. Immunohistochemical Analysis

The intestinal tissue sections were heated at 60 °C, deparaffinized, hydrated, repaired with antigen, extinguished with endogenous enzymes, and then blocked. The cells were then incubated with primary antibodies against Claudin 1 (Proteintech, Rosemont, IL, USA), Occludin (Proteintech, Rosemont, IL, USA), and ZO-1 (Proteintech, Rosemont, IL, USA). The sections were incubated with horseradish peroxidase-conjugated antibody, stained with 3,30-diaminobenzidine, counterstained with hematoxylin, and then blocked and observed. The protein expression levels of Claudin 1, Occludin, and ZO-1 were expressed as the average optical density (the ratio of integrated optical density to the tissue area) in at least six areas that were randomly selected for counting at 400× magnification as in a previous study [21].

### 2.8. Transmission Electron Microscopy (TEM)

After fixation with 3% glutaraldehyde, the tissue was postfixed in 1% osmium tetroxide, dehydrated in a series of acetone concentrations, infiltrated in Epox 812 for a longer period, and embedded. The semithin sections were stained with methylene blue, and ultrathin sections were cut with a diamond knife and stained with uranyl acetate and lead citrate. Sections were examined using a JEM-1400-FLASH Transmission Electron Microscope (JEOL, Tokyo, Japan) as described previously [22]. Two random photographs were taken for each ileal section, and the number of mitochondria per unit area (whole section) was counted.

### 2.9. Real-Time PCR Analysis

Total RNA was extracted from jejunum and ileum using Trizol (Beyotime, Shanghai, China), and the concentration and stability of isolated RNA were determined using NanoDrop 2000C Spectrophotometers (Thermo Fisher, Waltham, MA, USA). cDNA was synthesized using the Evo M-MLV reverse transcription kit (Accurate, Changsha, China). Each sample was evaluated thrice using the SYBR Green Premix Pro Taq HS qPCR Kit (Accurate, Changsha, China). The PCR procedure was as follows: 95 °C for 30 s, 95 °C for 5 s, and 60 °C for 30 s for 40 cycles. Relative mRNA expression levels were calculated using the 2^−ΔΔCt^ method and normalized to β-actin or GAPDH expression. The relative mRNA abundance of each gene was normalized to the piglets fed the basal diet and injected with saline. The primers used are listed in Table 2.

### 2.10. Statistical Analysis

Data were analyzed using SPSS (version 20.0; IBM-SPSS, Chicago, IL, USA), and GraphPad Prism (version 8.0; GraphPad Software, San Diego, CA, USA) was used for graphical processing. Statistical differences in the data were assessed using one-way analysis of variance, followed by Duncan’s multiple comparison test. Values of probability < 0.05 were used to denote statistically significant differences between the groups. Results are expressed as the mean ± standard error of the mean.

## 3. Results

### 3.1. Effect of Dietary GSH Supplementation at Different Levels on Growth Performance and Intestinal Morphology in Paraquat-Induced Weaned Piglets

**Figure 2:**
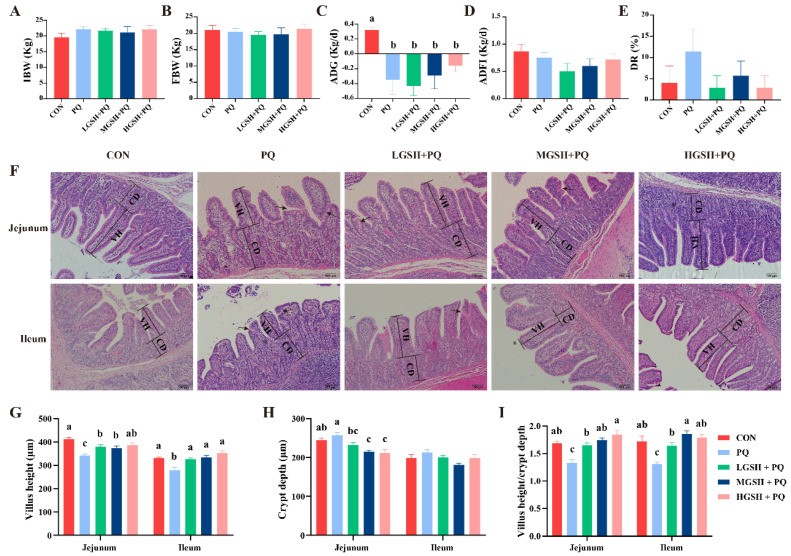
Effect of dietary GSH supplementation on growth performance and jejunal and ileal morphology in paraquat-induced weaned piglets. (**A**−**E**) The IBW (initial BW), FBW (final BW), ADG, ADFI, and DR for different groups. (**F**) Jejunal and ileal morphology of weaned piglets and representative images are shown (original magnifications, ×100; Scale bars = 100 µm). Black arrows indicate the site of damage. (**G**) Villus height of jejunum and ileum. (**H**) Crypt depth of jejunum and ileum. (**I**) Vellus height/crypt depth ratio of jejunum and ileum. Data values with different small letter superscripts indicate a significant difference (*p* < 0.05), while with the same or no letter superscripts indicate no significant difference (*p* > 0.05). Values are expressed as mean ± SEM, *n* = 7.

Under normal conditions, dietary GSH supplementation had no significant effects on the growth performance or DR of weaned piglets during four weeks (Figure 1B−F). However, compared with the CON group, the final BW, ADG, and ADFI in the HGSH group increased by 7.08%, 8.01%, and 11.54%, respectively, and had the greatest reduction in DR. In addition, the FCR in the MGSH group decreased. As shown in Figure 2, after the paraquat challenge, compared with the CON group, the ADG of the other groups was significantly decreased (*p* < 0.05); however, compared with the PQ group, the ADG in the HGSH + PQ group was slightly increased. There was no significant difference in ADFI or DR (*p* > 0.05).

Paraquat treatment led to jejunal and ileal morphological damage (marked with black arrows) in weaned piglets (Figure 2F). Dietary supplementation with different GSH concentrations markedly increased (*p* < 0.05) villus height and the villus height/crypt depth ratio in the jejunum and ileum of weaned piglets after paraquat treatment, and markedly decreased (*p* < 0.05) jejunal crypt depth (Figure 2G−I).

### 3.2. Effect of Dietary GSH Supplementation on Intestinal Permeability in Paraquat-Induced Weaned Piglets

**Figure 3:**
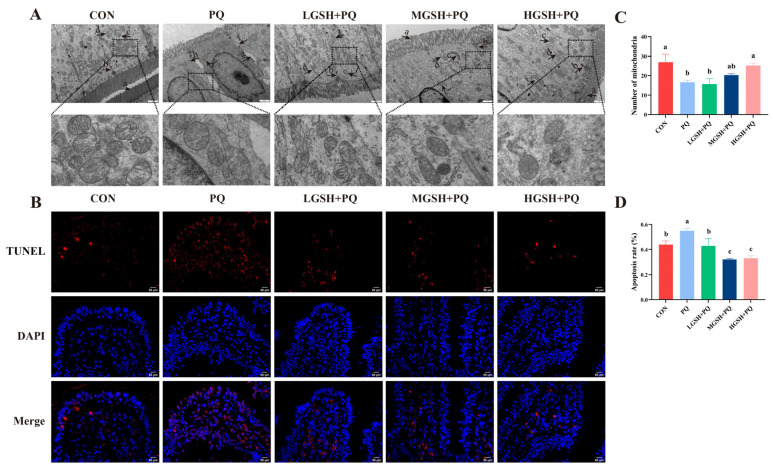
Effect of dietary GSH supplementation on ileal ultrastructure and apoptosis rate in paraquat-induced weaned piglets and representative images are shown. (**A**) Epithelial cells ultrastructure in the ileum. Original magnifications, 15,000×; Scale bars = 1 µm. Characteristic description: (a) regular microvilli. (b) clear tight junction. (c) normal mitochondria. (d) normal endoplasmic reticulum. (e) irregular microvilli. (f) tight connection is not clear. (g) wide intercellular space. (h) mitochondrial pyknosis. (i) mitochondrial swelling. (j) mitochondrial cristae abnormalities. (k) endoplasmic reticulum dilatation. (**B**) TUNEL staining of ileum (all cells shown in blue; apoptotic cells in red). Original magnification, ×400; Scale bars = 50 µm. (**C**) Number of mitochondria in ileal epithelial cells. (**D**) Quantitation of apoptosis rate in the ileum. Data values with different small letter superscripts indicate a significant difference (*p* < 0.05), while with the same or no letter superscripts indicate no significant difference (*p* > 0.05). Values are expressed as mean ± SEM, *n* = 7.

TEM images (Figure 3A,C) showed that, under paraquat treatment, the microvilli of piglets fed the basal diet were short and thick, loosely arranged, and morphologically irregular; tight junctions were not obvious, and cell gaps were widened; mitochondrial morphology was heterogeneous and significantly reduced in number (*p* < 0.05), with reduced and broken cristae; and endoplasmic reticulum appeared mildly dilated. Interestingly, GSH supplementation mitigated the intestinal ultrastructural injury caused by paraquat. The results of TUNEL staining showed that paraquat treatment led to a high level of apoptosis rate in the ileum of weaned piglets than in the CON group (Figure 3B), whereas the addition of GSH at different concentrations significantly reduced (*p* < 0.05) the apoptosis rate in the ileum (Figure 3D).

**Figure 4:**
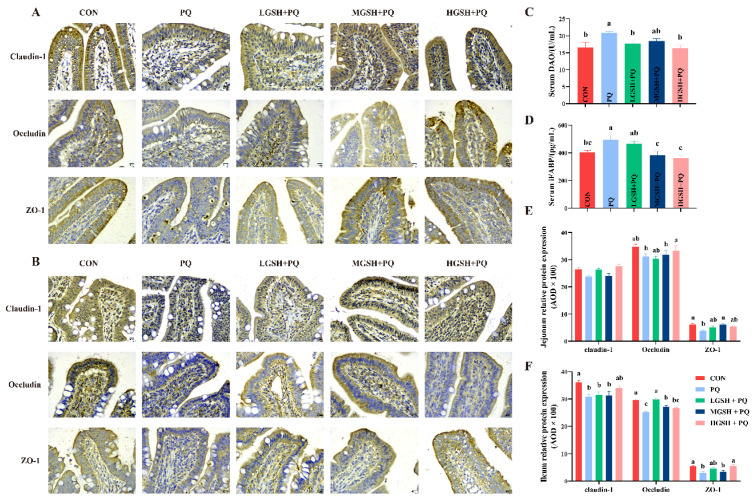
Effect of dietary GSH supplementation on intestinal tight junction proteins and permeability in paraquat-induced weaned piglets and representative images are shown. (**A**) Immunohistochemical staining of Claudin-1, Occludin, and ZO-1 in the jejunum (original magnifications, 400×; Scale bars = 50 µm). (**B**) Immunohistochemical staining of Claudin-1, Occludin, and ZO-1 in the ileum (original magnifications, 400×; Scale bars = 50 µm). (**C**,**D**) Serum iFABP and DAO levels. (**E**) The relative protein expression levels of Claudin-1, Occludin, and ZO-1 in the jejunum (AOD, average optical density). (**F**) The relative protein expression levels of Claudin-1, Occludin, and ZO-1 in the ileum (AOD, average optical density). Data values with different small letter superscripts indicate a significant difference (*p* < 0.05), while with the same or no letter superscripts indicate no significant difference (*p* > 0.05). Values are expressed as mean ± SEM, *n* = 7.

To investigate the effect of dietary GSH supplementation on intestinal barrier function in paraquat-induced weaned piglets, we determined the localization and expression of intestinal tight junction proteins, such as Occludin, Claudin-1, and ZO-1 proteins. The immunohistochemical results (Figure 4A,B,E,F) revealed that the ZO-1 expression level in the jejunum and Occludin, Claudin-1, and ZO-1 expression levels in the ileum of piglets injected with paraquat was significantly inhibited (*p* < 0.05); for oxidatively stressed piglets, GSH treatment significantly increased (*p* < 0.05) the expression levels of Occludin, and ZO-1 in the jejunum and ileum. After paraquat challenge, intestinal permeability was significantly changed (Figure 4C,D). Serum iFABP activity and DAO content in the PQ group were markedly higher (*p* < 0.05) than those in the CON group. As we envisioned, GSH supplementation reduced paraquat-induced (*p* < 0.05) intestinal permeability in piglets, with the best effect observed in the 0.06% GSH group.

### 3.3. Effect of Dietary GSH Supplementation on Antioxidant Capacity in Paraquat-Induced Weaned Piglets

**Figure 5:**
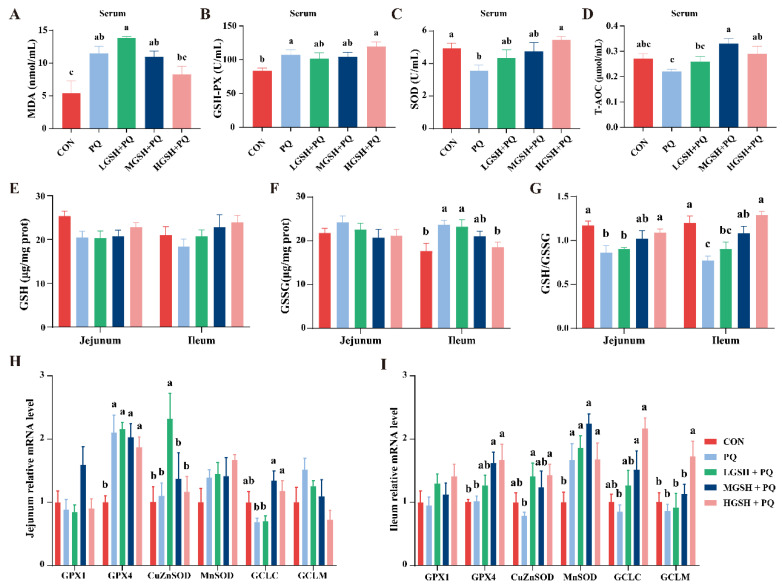
Effects of dietary GSH supplementation on antioxidant capacity in paraquat-induced weaned piglets. (**A**−**D**) MDA, GSH-PX, SOD, and T-AOC levels in serum. (**E**) GSH levels in the jejunum and ileum. (**F**) GSSG levels in the jejunum and ileum. (**G**) GSH/GSSG ratios in the jejunum and ileum. (**H**) The mRNA expression levels of *GPX1*, *GPX4*, *CuZnSOD*, *MnSOD*, *GCLC*, and *GCLM* in the jejunum. (**I**) The mRNA expression levels of *GPX1*, *GPX4*, *CuZnSOD*, *MnSOD*, *GCLC*, and *GCLM* in the ileum. Data values with different small letter superscripts indicate a significant difference (*p* < 0.05), while with the same or no letter superscripts indicate no significant difference (*p* > 0.05). Values are expressed as mean ± SEM, *n* = 7.

To evaluate whether dietary GSH supplementation could alleviate oxidative stress injury in weaned piglets, the levels of antioxidant-related enzymes in the serum and small intestine were measured. Compared with the CON group, paraquat challenge caused a significant increase (*p* < 0.05) in serum MDA content (Figure 5A), GSH-PX activity (Figure 5B), and ileal GSSG content (Figure 5F) but markedly decreased (*p* < 0.05) serum SOD activity (Figure 5C) and jejunal and ileal GSH/GSSG ratios (Figure 5G). In contrast, compared with the PQ group, the addition of 0.06% GSH to the diet significantly decreased (*p* < 0.05) the ileal GSSG level (Figure 5F) but significantly increased (*p* < 0.05) serum T-AOC content (Figure 5D), SOD activity, and GSH/GSSG ratios in the jejunum and ileum. The serum T-AOC content and ileal GSH/GSSG ratio in the MGSH+PQ group were significantly higher (*p* < 0.05) than those in the PQ group. Nevertheless, there was no obvious change in GSH content in the jejunum and ileum (*p* > 0.05) (Figure 5E). The mRNA expression levels of jejunal *GPX4* and ileal *MnSOD* were significantly increased (*p* < 0.05) in the PQ group compared with those in the CON group (Figure 5H,I). Dietary GSH supplementation significantly increased (*p* < 0.05) the mRNA expression levels of jejunal *CuZnSOD* and *GCLC* and ileal *GPX4*, *CuZnSOD*, *GCLC*, and *GCLM* (Figure 5H,I).

### 3.4. Effect of Dietary GSH Supplementation on the Expression of Inflammatory Cytokine and CAR Pathway-Related Targets in Paraquat-Induced Weaned Piglets

**Figure 6:**
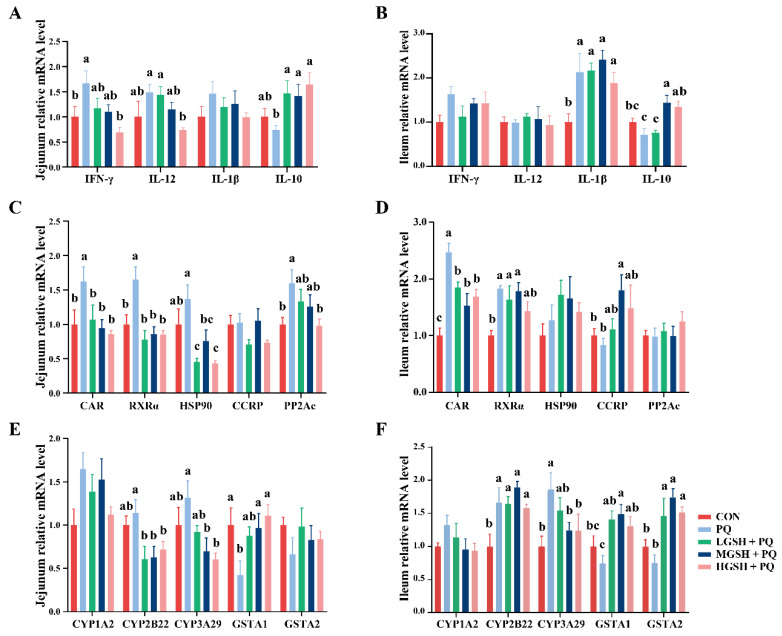
Effects of dietary GSH supplementation on the expression of inflammatory cytokines and CAR-regulated pathway targets in paraquat-induced weaned piglets. (**A**,**B**) The mRNA expression levels of *IFN-γ*, *IL-12*, *IL-1β*, and *IL-10* in the jejunum and ileum. (**C**,**D**) The mRNA expression levels of *CAR*, *RXRα*, *HSP90*, *CCRP*, and *PP2Ac* in the jejunum and ileum. (**E**,**F**) The mRNA expression levels of *CYP1A2*, *CYP2B22*, *CYP3A29*, *GSTA1*, and *GSTA2* in the jejunum and ileum. Data values with different small letter superscripts indicate a significant difference (*p* < 0.05), while with the same or no letter superscripts indicate no significant difference (*p* > 0.05). Values are expressed as mean ± SEM, *n* = 7.

Oxidative stress is generally accompanied by an inflammatory response; we investigated whether GSH can regulate the gene expression of inflammatory cytokines in the jejunum and ileum. As shown in Figure 6A,B, the mRNA expression levels of jejunal *IFN-γ* and *IL-12* in the HGSH+PQ group were significantly lower (*p* < 0.05) than those in the PQ group. Moreover, GSH treatment upregulated (*p* < 0.05) *IL-10* mRNA expression in the jejunum and ileum of paraquat-induced weaned piglets.

To investigate whether GSH could protect intestinal tissues from oxidative stress by regulating the CAR pathway, we examined the key targets of the CAR pathway in the intestine. Compared with the PQ group, the addition of GSH at different concentrations in the diets dramatically decreased (*p* < 0.05) the mRNA expression levels of *CAR*, *RXRα*, *HSP90*, and *PP2Ac* (Figure 6C) and dramatically decreased (*p* < 0.05) the mRNA expression levels of the target genes *CYP2B22*, and *CYP3A29*, while increasing (*p* < 0.05) the mRNA expression level of the target gene *GSTA1* in the jejunum (Figure 6E). In the ileum, GSH supplementation similarly decreased (*p* < 0.05) the mRNA expression levels of *CAR* and *CYP3A29* and increased (*p* < 0.05) the mRNA expression levels of *CCRP*, *GSTA1*, and *GSTA2* (Figure 6D,F).

## 4. Discussion

The small intestine is an organ in which the host dynamically interacts with the intestinal luminal environment and is extremely vulnerable to oxidative stress [23]. If the body continues to generate high levels of ROS for a long time, it can cause irreversible damage to the cell structure and function, which, in turn, leads to cell necrosis and apoptosis, endangering the health of the body [24]. Paraquat has been widely used as a method to induce acute oxidative stress in mammals, as previously described [25,26,27]. In the current study, we also used paraquat to obtain an oxidative stress-weaned-piglet model. Our results showed that, under normal conditions, dietary supplementation with different GSH concentrations had no significant effect on the growth performance or DR of weaned piglets. The findings are similar to those previously reported [28]. The reasons for the unchanged growth performance in a normal situation may be related to the short experimental period and the small number of experimental samples. After the paraquat challenge, the ADG of each group was significantly reduced; however, the addition of 0.06% GSH to the diet reversed this reduction, indicating that 0.06% GSH may improve the antioxidant ability to resist stress. In addition, we observed that exogenous GSH reduced the DR and increased intestinal villus height in oxidative stress-induced-weaned piglets. The higher villus and shallower crypt indicate a higher digestive and absorptive capacity of the intestine and a faster cell maturation rate [29], which may predict that exogenous GSH is associated with an improved intestinal barrier function. We believe that a part of the exogenous GSH is degraded into its synthetic substrates (glutamate, glycine, and cysteine), which can stimulate GSH synthesis in vivo and play a functional role, whereas the other part is directly absorbed and utilized by the body, thus increasing nutrient metabolism, antioxidant capacity, and immune response.

The intestinal barrier function is an important basis for assessing the health status of the intestine. An osmotic barrier that selectively allows the passage of nutrients and water while resisting the effects of bacteria, toxins, and harmful flora in the intestinal lumen plays a key role in the regulation of the immune system [30,31]. In the present study, dietary supplementation with GSH improved the intestinal morphology of paraquat-induced weaned piglets and inhibited the apoptosis of ileal epithelial cells, which suggests its role in cell renewal and proliferation. Excess ROS produced by oxidative stress leads to apoptosis and ferroptosis, whereas lipid peroxidation induced by ferroptosis causes organelle damage such as plasma membrane rupture, endoplasmic reticulum expansion, and mitochondrial morphological changes [32]. Our results revealed that dietary GSH supplementation partially alleviated paraquat-induced mitochondrial and endoplasmic reticulum damage. Similar to previous studies, the depletion of GSH in vivo disrupts the structural integrity of the mitochondria [33]. These results suggest that dietary supplementation with GSH exerts a protective effect on the intestinal structure of weaned piglets by attenuating organelle damage and apoptosis. This may be related to exogenous GSH improving mitochondrial ATP synthesis and energy metabolism and enhancing the antioxidant capacity of the organism.

Tight junction proteins, which are essential components of the intestinal barrier, regulate intestinal permeability [34]. In this study, we found that Claudin-1, Occludin, and ZO-1 proteins were mainly located in the intestinal epithelial cell membrane, and paraquat challenge disrupted their original distribution and expression. This may be relevant to the redistribution of nutrient transport channel proteins, which is similar to the study by He et al. (2020), who found that Gln, an effective precursor for the synthesis of GSH, improves nutrient transport carrier activity and tight junction permeability [20]. The disruption of tight junctions leads to various cellular dysfunctions, damage to the intestinal barrier, and increased permeability, which can promote diseases such as inflammatory bowel disease [35,36]. However, dietary GSH supplementation attenuated this damage, with similar results observed by TEM images. Serum iFAPB and DAO activities are also important indicators of intestinal permeability [37,38]. Diet supplemented with GSH could reduce the significant elevation of serum iFAPB and DAO activity caused by paraquat, which reduces intestinal permeability. These results illustrate that dietary supplementation with GSH modulates oxidative stress-induced disruption of intestinal structure and changes in barrier regulatory components, improves intestinal barrier function, and restores intestinal health.

To avoid the damage caused by oxidative stress, two antioxidant defense systems exist in the organism: enzymatic antioxidant systems, including SOD, GSH-PX, and CAT, and non-enzymatic antioxidant systems, including GSH, VC, VE, and trace elements (e.g., Cu, Zn, and Se) [39]. Our results revealed that dietary supplementation with GSH increased T-AOC, SOD activity, and GSH/GSSG ratios and decreased MDA and GSSG levels. Second, exogenous GSH upregulated the mRNA expression of *CuZnSOD*, *GCLC*, and *GCLM* in the jejunum and ileum. These results revealed that exogenous GSH enhanced endogenous GSH synthesis by up-regulating the expression of the rate-limiting enzyme gene (GCLC/M) during GSH biosynthesis, which, in turn, affects the activity of other antioxidant enzymes to improve antioxidant capacity. Furthermore, oxidative stress and inflammatory responses are interrelated, and oxidative stress damage may trigger inflammatory processes, whereas pro-inflammatory factors produce more ROS/RNS [40,41]. Our study found that exogenous GSH attenuated the inflammatory response triggered by paraquat, correspondingly inhibiting the gene expression of the pro-inflammatory factors *IFN-γ* and *IL-12* and promoting the gene expression of the anti-inflammatory factor *IL-10*. These results indicate that exogenous GSH supplementation in vivo may evert a protective effect against paraquat-induced intestinal injury, and the underlying mechanism is likely related to the suppression of oxidative stress and inflammatory response.

To further elucidate this mechanism, we investigated CAR signaling pathway expression. In its natural state, CAR is retained in the cytoplasm mainly through cytoplasmic CAR retention protein (CCRP)-mediated binding to HSP70/90 to form the CAR-CCRP-HSP70/90 complex. Protein phosphatase 2A (PP2A) dephosphorylates CAR and translocates it to the nucleus, where it binds to RXRα to form a heterodimer, which in turn regulates the transcription and expression of downstream target genes [42,43]. Our results showed that paraquat treatment upregulated the mRNA expression levels of *CAR*, *RXRα*, and *PP2Ac*, but exogenous GSH reversed this effect. The activation of CAR may be crucial in xenobiotic metabolism and oxidative stress, which is consistent with the results of Yoda et al. (2017), who found that oral administration of phenethyl isothiocyanate to mice resulted in oxidative stress generation, upregulating the protein expression of UGT1A1 and Cyp2b10, which was mediated by CAR, whereas N-acetylcysteine suppressed this phenomenon [17]. CAR can also regulate metabolic and immune processes by modulating downstream effector target genes (e.g., CYP2B, CYP3A, CYP1A, GSTA1, and GSTA2) [44,45]. We then examined the expression of downstream target genes and found that exogenous GSH downregulated the expression levels of *CYP2B22* and *CYP3A29* and upregulated the expression of *GSTA1* and *GSTA2* genes by activating CAR. GSTs bind to and react with various electrophilic xenobiotics, cellular metabolites, environmental pollutants, and drugs to form sulfhydryl compounds that exert detoxifying effects [46]. The above findings suggest that dietary supplementation with GSH improves piglet intestinal health by inhibiting paraquat-induced activation of the intestinal CAR signaling pathway while reducing oxidative stress and the inflammatory response.

## 5. Conclusions

In conclusion, our results demonstrated that dietary supplementation with GSH partially improved growth performance and reduced the frequency of diarrhea in paraquat-induced weaned piglets. Exogenous GSH administration enhanced the activity and mRNA expression of serum and intestinal antioxidant-related enzymes, inhibited cytokine secretion, reduced the inflammatory response, and improved intestinal structure, tight junction proteins, intestinal permeability, and apoptosis by regulating the CAR signaling pathway, thereby alleviating oxidative stress damage. Our study provides a theoretical basis for exploring the correlations among gut health, oxidative stress damage, and the corresponding treatment strategies. Future research will delve into the role of GSH in mitochondrial function and the specific ways in which the CAR signaling pathway functions under stress conditions.

## Figures and Tables

**Table 1:** Composition and nutrient levels of basal diets (air-dry basis, %).

Ingredients	Content	Nutrient Level ^2^	Content
Ripening corn	31.45	Net energy (MJ/KG)	24.50
Ripening rice	25.60	Crude protein	17.20
Flour	12.50	Ether extract	3.70
Extruded soybean	6.40	Crude fiber	2.08
Fish meal	3.50	Calcium	0.59
Ripening soybean meal	5.00	Phosphorus	0.51
Fermented soybean meal	5.00	Digestible Lysine	1.30
50% Choline chloride	0.10	Digestible Methionine	0.59
CaHPO3	0.80	Digestible Methionine + Cystine	0.80
Limestone	0.50	Digestible Tryptophan	0.27
Glucose	2.50	Digestible Threonine	0.85
Sucrose	2.50		
Soybean oil	1.25		
NaCl	0.40		
Lysine HCL	0.77		
Threonine	0.36		
Methionine	0.38		
L-Tryptophan	0.12		
L-Valine	0.38		
Premix ^1^	0.19		
Total	100.00		

Note: ^1^ The premix provided the following per kg of the diet: vitamin A, 10,000 IU; vitamin D, 3000 IU; vitamin E, 30 mg; vitamin K3, 4 mg; vitamin B1, 4 mg; vitamin B2, 10 mg; vitamin B6, 6 mg; vitamin B12, 0.04 mg; nicotinic acid, 40 mg; pantothenic acid, 20 mg; folic acid, 2 mg; biotin, 0.2 mg; Cu (as copper sulfate pentahydrate), 25 mg; Fe (as ferrous sulfate), 100 mg; Mn (as manganese sulfate), 25 mg; Zn (as zinc sulfate), 80 mg; I (as Calcium iodate), 0.8 mg; Se (as sodium selenite), 0.35 mg; and Co (as cobalt chloride), 0.5 mg. ^2^ Nutrient levels were calculated values.

**Table 2:** Primers used for quantitative reverse transcription-PCR.

Gene	Primers	Accession Numbers	Product Length (bp)
GPX1	F: TGGGGAGATCCTGAATTG R: GATAAACTTGGGGTCGGT	NM_214201.1	184
GPX4	F: GATTCTGGCCTTCCCTTGC R: TCCCCTTGGGCTGGACTTT	NM_214407.1	173
MnSOD	F: GGACAAATCTGAGCCCTAACG R: CCTTGTTGAAACCGAGCC	NM_214127.2	159
CuZnSOD	F: TGAAGGGAGAGAAGACAGTGTTAG R: TCTCCAACGTGCCTCTCTTG	NM_001190422.1	181
GCLC	F: GATCCTCCAGTTCCTGCACA R: GAGAGAGAACCAACCTCGTCG	XM_021098556.1	87
GCLM	F: CACAGCGAGGAGCTTCGAGA R: TGCGTGAGACACAGTACATTCC	XM_001926378.4	117
IFN-γ	F: CAGGCCATTCAAAGGAGCAT R: GAGTTCACTGATGGCTTTGCG	NM_213948.1	150
IL-1β	F: CCAATTCAGGGACCCTACCC R: GTTTTGGGTGCAGCACTTCAT	NM_214055.1	174
IL-12	F: CAGGCCCAGGAATGTTCAAA R: CGTGGCTAGTTCAAGTGGTAAG	NM_213993.1	188
IL-10	F: CGGCGCTGTCATCAATTTCTG R: CCCCTCTCTTGGAGCTTGCTA	NM_214041.1	89
CAR	F: GTGCCTGAACTGTCTCTGCT R: CCACATGCGCTCCATCTTCT	NM_001037996.1	244
RXRα	F: CAAGTGCCTGGAACACCTCT R: ATGGAAGGTAACAGGGTGGC	XM_001927453.2	240
HSP90	F: AAGACCGGACCCTCACGATA R: AGGCATACTGCTCGTCATCG	NM_213973.1	231
CCRP	F: TGCCCTAGAATTTGCCCCTG R: GCAAAGACCTCGGACGTACA	XM_003131409.5	157
PP2Ac	F: GGTGCCATGACCGGAATGTA R: GTGCTGGGTCAAACTGCAAG	NM_214366.1	129
GSTA1	F: AGGACACCCAGGACCAATCTT R: CTCAGGTACATTCCGGGAGAAG	NM_214389.2	199
GSTA2	F: CTACTACGTGGAAGAGCTGGAC R: GCCCTGCCCACTTTATGAAGAC	NM_213850.2	193
CYP1A2	F: TTTGTGGAGACCGCCTCATC R: GCTTGAATAGGGCGCTTGTG	NM_001159614.1	193
CYP2B22	F: GGGAACGTTGGAAGACCCTT R: CGGGATCTCTGTAGGCGAAG	NM_214413.1	228
CYP3A29	F: CCTGAAATTAACCACGCAAGGGCT R: TCTGGGATGCAGCTTTCTTGACCA	NM_214423.1	140
β-actin	F: CTGCGGCATCCACGAAACT R: AGGGCCGTGATCTCCTTCTG	XM_003124280.3	147
GAPDH	F: AAGGAGTAAGAGCCCCTGGA R: TCTGGGATGGAAACTGGAA	NM_001206359.1	140

## Data Availability

The data used to support the findings of this study are available from the corresponding author upon request.

## Abbreviations

Paraquat (PQ); Glutathione (GSH); Reactive oxygen species (ROS); Cytoplasmic CAR retention protein (CCRP); Heat shock protein 90 (HSP90); Constitutive androstane receptor (CAR); Retinoid X receptor alpha (RXRα); Protein phosphatase 2A catalytic subunit (PP2Ac); Cytochrome P450 (CYP); Xenobiotic responsive enhancer modules (XREMs).
